# Insights into intercellular receptor-ligand binding kinetics in cell communication

**DOI:** 10.3389/fbioe.2022.953353

**Published:** 2022-06-28

**Authors:** Chenyi An, Xiaohuan Wang, Fan Song, Jinglei Hu, Long Li

**Affiliations:** ^1^ School of Biology and Engineering, Guizhou Medical University, Guiyang, China; ^2^ Department of Cell Biology and Department of Cardiology of the Second Affiliated Hospital, Zhejiang University School of Medicine, Zhejiang University, Hangzhou, China; ^3^ Department of Rehabilitation Medicine, Peking University Third Hospital, Beijing, China; ^4^ State Key Laboratory of Nonlinear Mechanics and Beijing Key Laboratory of Engineered Construction and Mechanobiology, Institute of Mechanics, Chinese Academy of Sciences, Beijing, China; ^5^ School of Engineering Science, University of Chinese Academy of Sciences, Beijing, China; ^6^ Kuang Yaming Honors School and Institute for Brain Sciences, Nanjing University, Nanjing, China

**Keywords:** cell communication, intercellular receptor-ligand binding kinetics, protein-membrane interaction, biomechanical force, bioelectric microenvironment

## Abstract

Cell-cell communication is crucial for cells to sense, respond and adapt to environmental cues and stimuli. The intercellular communication process, which involves multiple length scales, is mediated by the specific binding of membrane-anchored receptors and ligands. Gaining insight into two-dimensional receptor-ligand binding kinetics is of great significance for understanding numerous physiological and pathological processes, and stimulating new strategies in drug design and discovery. To this end, extensive studies have been performed to illuminate the underlying mechanisms that control intercellular receptor-ligand binding kinetics *via* experiment, theoretical analysis and numerical simulation. It has been well established that the cellular microenvironment where the receptor-ligand interaction occurs plays a vital role. In this review, we focus on the advances regarding the regulatory effects of three factors including 1) protein-membrane interaction, 2) biomechanical force, and 3) bioelectric microenvironment to summarize the relevant experimental observations, underlying mechanisms, as well as their biomedical significances and applications. Meanwhile, we introduce modeling methods together with experiment technologies developed for dealing with issues at different scales. We also outline future directions to advance the field and highlight that building up systematic understandings for the coupling effects of these regulatory factors can greatly help pharmaceutical development.

## Introduction

Cells communicate with their neighbors to sense, respond and adapt to outside world, and transduce crucial signals to shape their functions and determine their fate. The cell-cell communication process, which involves multiple length scales ranging from angstroms (specific binding of receptors and ligands), to tens of nanometers (length of receptor-ligand complex) to micrometers (lateral size of a typical adhesion zone), is mediated by the specific binding of receptor and ligand anchored in apposing membranes (Liu et al., 2014; Ju et al., 2016; Sudhof, 2018; Zhu et al., 2019a; Zhu et al., 2019b; Chen et al., 2019). The key parameters characterizing the intercellular binding of receptors and ligands are their binding kinetics that involve kinetic rates (on-rate, *k*_on_ and off-rate, *k*_off_, defining the velocities of bond formation and dissociation, respectively) and binding affinity (*K*_a_ = *k*_on_ / *k*_off_ = [RL] / ([R][L]), quantifying the strength of receptor-ligand interactions) (Hu et al., 2013; Liu et al., 2015). Here, [R], [L] and [RL] are densities of unbound receptors, unbound ligands and bound receptor-ligand complexes in equilibrium state, respectively. The binding kinetics of intercellular receptor-ligand interactions determine the extent of membrane receptors’ transmembrane signaling during cell communication and thereby affect physiological and pathological cellular activities, such as immune responses, cell locomotion and cancer metastasis (Burdick et al., 2001; Huang et al., 2010; Chen and Zhu, 2013; Liu et al., 2014; Liu et al., 2015). For example, the binding kinetics of interactions between T cell receptor (TCR) and peptide major histocompatibility complex (pMHC) determine the recognition of T cells to different antigens and the processes of target cell killing, with accumulated receptor-ligand binding duration as a threshold for triggering T cells activation (Huang et al., 2010; Liu et al., 2014; Wu et al., 2019). Mutations in von Willebrand factor (VWF) and/or platelet glycoprotein Ib (GPIb), which alter their binding kinetics, induce hemostatic defects, such as von Willebrand disease (Ju et al., 2013). Considering the vital role of intercellular receptor-ligand binding kinetics in processing extracellular stimuli to direct cellular activities and their promising potential for biomedical applications, such as immunotherapies including monoclonal antibodies, chimeric antigen receptor T (CART) cells and TCR-T cells, the regulatory mechanisms responsible for the receptor-ligand binding kinetics and their transmembrane signaling have been hotspots in the fields of mechanobiology (Fagerberg et al., 2010; An et al., 2020; Li R. et al., 2020b; He et al., 2022).

Much of our early understanding of receptor-ligand binding kinetics came from *in vitro* three-dimensional (3D) measurements by surface plasmon resonance (SPR) for purified variants of the receptors and ligands that are removed from their cellular environment (Huang et al., 2012). The kinetics parameters are derived by SPR angle shifts when the mass of the surface layer changes due to receptor-ligand binding (Su and Wang, 2018). However, there are significant limitations for SPR in faithfully investigating receptor-ligand interactions due to the lack of physiological-mimicking conditions (Huang et al., 2010). For example, compared to *in vitro* 3D measurements in solution, the *in situ* receptor-ligand binding occurs in two dimensions (2D) with both proteins anchored in apposing membranes, resulting in different units for on-rate *k*_on_ (M^-1^ s^-1^ in 3D and μm^2^ s^-1^ in 2D) and binding affinity *K*_a_ (M^-1^ in 3D and μm^2^ in 2D) in different dimensions (Dustin et al., 2001). Thus, the binding kinetics measured by SPR cannot be used to derive reliable information on 2D binding. With the development of technology, various experimental techniques have been exploited to study the intercellular receptor-ligand binding kinetics, including fluorescence spectroscopy, micropipette aspiration, atomic force microscopy, and flow chamber (Krobath et al., 2009; Weikl et al., 2016). It has been well established that the *in situ* kinetics of receptor-ligand interactions depend not only on the receptor-ligand binding strength, but also on the cellular microenvironment. For example, utilizing live-cell based single-molecule biomechanical assay, Fan et al. showed that the bond lifetimes (1 / *k*_off_) of stimulatory immune receptor NKG2D interacting with its ligands (e.g., MICA and MICB) are prolonged in the presence of mechanical force (Fan et al., 2022). The force-dependent bond lifetime and binding affinity are attributed to the conformational changes of ligands, enabling NKG2D to precisely discriminate ligands to differentially activate natural killer cell or T cell functions and fulfill proper immune responses (Fan et al., 2022). Chen et al. used a fluorescence dual biomembrane force probe to identify an intermediate state of integrin αIIbβ_3_ with intermediate affinity and bond lifetimes by applying precisely controlled mechanical stimulations to platelets (Chen et al., 2019). They found that this intermediate state of integrin αIIbβ_3_ regulates biomechanical platelet aggregation, which is responsible for the thrombus formation and growth. These studies provide new insights into the intercellular receptor-ligand binding kinetics in cell communication, and promising therapeutic strategies for the disease treatment. In this review, we summarize recent advances regarding the regulation of intercellular receptor-ligand binding kinetics by three *in situ* membrane-associated factors, including 1) protein-membrane interaction, 2) biomechanical force, and 3) bioelectric microenvironment, wherein modeling approaches and experiment technologies used to deal with issues at different scales are introduced. Further, we outline directions for dissecting functional mechanisms of intercellular receptor-ligand binding and indicate optimization strategies for biomedical applications.

## Protein-membrane interaction

In contrast to the interactions of soluble proteins in solution, the interactions of membrane-anchored receptors and ligands during cell-cell communication are restricted to two-dimensional membrane environment, since the proteins can only diffuse laterally along the membranes (Krobath et al., 2009; Hu et al., 2013; Weikl et al., 2016). Central questions involve how the binding kinetics of membrane-anchored receptor and ligand are affected by protein-membrane interaction, which will be discussed in this section.

### Membrane fluctuations

A membrane-anchored receptor can bind to a ligand anchored in apposing membrane only if the local membrane separation at the protein site is within binding range. Therefore, the binding of receptor and ligand molecules depends strongly on the local separation of the two membranes, which varies in time and space due to the thermal shape fluctuations of flexible membranes (Li and Song, 2016; Li and Song, 2018). As mentioned above, the intercellular communication process involves multiple length scales ranging from angstroms to micrometers. To deal with such complexity, coarse-grained mechanical models have been developed with suitable simplification and approximations (Krobath et al., 2009; Rozycki et al., 2010; Hu et al., 2013; Xu et al., 2015; Weikl et al., 2016). In the Monte Carlo (MC) simulation model, both adhering membranes are discretized into small patches (Krobath et al., 2009; Xu et al., 2015). Then the membrane conformations can be described by the local intermembrane separation. By analogy to lattice-gas-type models, a membrane patch can only accommodate one receptor or ligand protein. The spatial distribution of receptor and ligand is then described by the composition field with values 0 or 1 indicating the absence or presence of protein at discretized patch. The membrane anchoring of receptor and ligand, which can rotate around their anchoring points, is regulated by the protein-membrane anchoring strength. To reflect the protein flexibility, the bead-spring model for polymer chains can be adopted. The specific binding of receptor and ligand shows a distance- and angle-dependent behavior. The configurational energy of the adhesion system then consists of membrane bending energy, receptor-ligand binding energy, as well as anchoring energy. This MC model basically captures the key events that occur in the adhesion zone. Another coarse-grained model is based on molecular dynamics (MD) technique (Hu et al., 2013; Hu et al., 2015). In the coarse-grained MD simulation model, several molecule groups are clustered into a single bead. This coarse graining procedure leads to a significant computational speed-up. Each lipid molecule, receptor and ligand proteins are represented by a set of beads connected *via* spring potential. A lipid molecule consists of hydrophilic and hydrophobic parts. Both lipid-anchored and transmembrane receptor and ligand proteins can be modeled. The thermodynamic properties of the adhesion system are determined by the conservative force. In comparison, the coarse-grained MC method has advantages in both computing scale and efficiency. Results from statistic mechanics theory and coarse-grained simulations indicate that the binding affinity and on-rate can be obtained from the relations: $K_{\text{a}}=\int K_{\text{a}}\left(l\right)P\left(l\right)dl$ , $k_{\text{on}}=\int k_{\text{on}}\left(l\right)P\left(l\right)dl$ , where *K*_a_ (*l*) and *k*_on_ (*l*) are the binding affinity and on-rate of receptor and ligand anchored to two planar and parallel membranes with fixed separation *l*, respectively (Hu et al., 2015; Weikl, 2018). The probability distribution of the local membrane separation *P*(*l*) reflects the temporal and spatial variation of local membrane separation *l* and is associated with the membrane elasticity. For adhering membranes with a single type of receptor-ligand complex, this probability distribution *P*(l) is usually assumed to be Gaussian with mean $\bar{l}=l$ and standard deviation $\xi_{⊥}=\sqrt{{\left(l-\bar{l}\right)}^{2}}$ (Hu et al., 2015). $\xi_{⊥}$ is the relative roughness from thermally excited shape fluctuations and measures how strong the fluctuations of the two membranes are (Hu et al., 2013; Li et al., 2018b). The larger the roughness $\xi_{⊥}$ , the stronger the membrane fluctuations and the more configurations the membranes can adopt. The formation of receptor-ligand complexes constrains the membrane thermal fluctuation, thus affecting the relative roughness. The relative roughness is proportional to the average distance between neighboring receptor-ligand complex $\xi_{⊥}∼1/\sqrt{\left[\text{RL}\right]}$ (Hu et al., 2013; Weikl, 2018).

At the optimal average membrane separation for receptor-ligand binding, theoretical and computational results consistently reveal that the binding affinity roughly scales as: $K_{\text{a}}\;∼1/\xi_{⊥}$ (Hu et al., 2013). Such an inverse proportionality results from the entropy loss of the flexible membranes upon the receptor-ligand binding. Coarse-grained MD simulations show that the on-rate *k*_on_ and off-rate *k*_off_ decrease and increase with increasing relative roughness, respectively, indicating that both *k*_on_ and *k*_off_ contribute to the roughness-dependence of *K*_a_. Given the scaling relation $\xi_{⊥}∼1/\sqrt{\left[\text{RL}\right]}$ , a modified law of mass action $\left[\text{RL}\right]\;∼{\left[\text{R}\right]}^{2}{\left[\text{L}\right]}^{2}$ appears (Krobath et al., 2009; Hu et al., 2013). This quadratic dependence indicates cooperative binding of membrane adhesion proteins. The physical picture is that the formation of the receptor-ligand complexes suppresses membrane shape fluctuations which, in turn, facilitates the formation of additional receptor-ligand complexes. The feedback between the suppression of membrane fluctuations and the formation of receptor-ligand complexes gives rise to cooperativity in the process of receptor-ligand binding (Li et al., 2021b). Recently, Steinkühler et al. confirmed the binding cooperativity of ‘marker of self’ protein CD47 with the macrophage checkpoint receptor SIRPα using fluorescent recovery after bleaching (FRAP) assays, and found that membrane stiffening by regulating acidosis condition enhances the binding affinity (Steinkühler et al., 2019).

Different experimental methods for measuring the binding affinity have led to values differing by several orders of magnitude (Krobath et al., 2009). Note that mechanical methods measure the receptor-ligand binding kinetics during initial contacts. In contrast, fluorescence methods probe the binding kinetics in equilibrated zones (Weikl et al., 2016). Considering the fact that both the average membrane separation $\bar{l}$ and relative membrane roughness $\xi_{⊥}$ during initial membrane contacts are larger than that in equilibrated adhesion zones, the dependence of *K*_a_ on $\bar{l}$ and $\xi_{⊥}$ helps to understand why the values of *K*_a_ measured by mechanical and fluorescence methods differ by several orders of magnitude.

### Protein-Lipid Interaction

**FIGURE 1:**
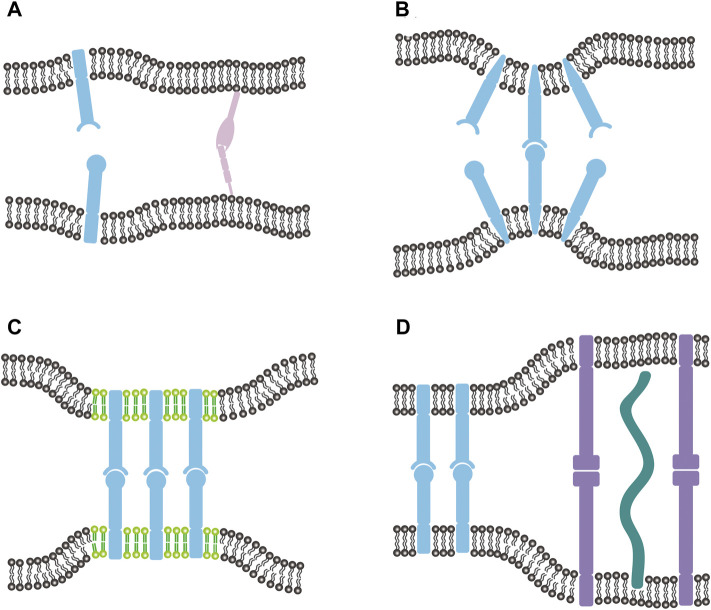
**(A)** Two fluctuating membranes adhering *via* specific binding of transmembrane (light blue) and lipid-anchored (light purple) receptor and ligand proteins. Both the thermal shape fluctuations of membranes, which change the average membrane separation and relative membrane roughness, and the anchoring energy affect the receptor-ligand binding kinetics. **(B)** Wedge-shaped transmembrane proteins bend their associated membranes to induce membrane curvature. The local curvatures induced by receptor and ligand affect their binding kinetics by 1) altering the local separation and relative roughness of the two apposing membranes and 2) causing protein-protein *cis*-repulsion on each membrane. **(C)** Preferential partitioning of membrane-anchored receptors and ligands in the lipid rafts (light green) enhances the binding affinity of those proteins, which can be partially attributed to the entropy gain of the membranes resulting from raft-induced protein aggregation. **(D)** Adhering membranes linked *via* two types of membrane-anchored receptors and ligands with different lengths in the presence of glycocalyx (dark green). The difference in lengths between the shorter and longer receptor-ligand complexes, on the one hand, forms a steric barrier for the complex formation, on the other hand, facilitates protein aggregation to enhance binding. These two competing effects are additionally regulated by the presence of glycocalyx.

The membrane proteins associate with cell membranes *via* transmembrane domains (e.g. integrins, cadherins) or glycosylphosphatidyl-inositol anchors (e.g. CD48). The anchoring to membranes is of particular importance for intercellular receptor-ligand interaction and protein function (Figure 1A). For example, an I232T mutation in Fcγ receptor, which is clinically relevant to systemic lupus erythematosus, alters the interaction between transmembrane helix of Fcγ receptor and membrane. As a result, the mutated Fcγ receptor allosterically tilts its ectodomain to a bent conformation, which attenuates its accessibility by ligands and thereby reduces its ligand-binding affinity (Hu et al., 2019). Meanwhile, the anchoring strength, characterizing the tilting of binding protein relative to the membrane, also plays an important role in the binding kinetics of receptor and ligand molecules, since it affects the loss in the rotational free energy upon the formation of a receptor-ligand complex. Therefore, soft anchoring of binding proteins decreases binding affinity and slower on-rate (Hu et al., 2015; Xu et al., 2015).

The interaction of protein and membrane may very likely generate local membrane curvature and accordingly affect the intercellular receptor-ligand interactions. Extensive studies have shown that transmembrane proteins of wedge shape, and peripheral proteins either inserting asymmetric amphipathic or hydrophobic structures into the bilayer or binding to the surface of one membrane monolayer are efficient ways to induce local membrane curvature (Figure 1B) (McMahon and Gallop, 2005; Ramakrishnan et al., 2014; McMahon and Boucrot, 2015). Crowding of monomeric hydrophilic protein domains bound to the membrane surface has also been shown to induce curvature (Stachowiak et al., 2012). MC simulations revealed that the local membrane curvatures induced by receptors and ligands affect their binding by 1) altering the local separation and relative roughness of the two adhering membranes, and 2) causing protein-protein *cis*-repulsion on each membrane (Li et al., 2019). Depending on the signs of the curvatures, the binding affinity increases or decreases with the curvatures. It suggests that the ability to induce membrane curvatures represents a molecular property of the adhesion proteins and should be carefully considered in experimental characterization of the binding affinity.

Local aggregation or clustering of the protein molecules also affects their binding kinetics. Cumulative evidence indicates that the membranes are not structurally homogeneous, but rather consist of microdomains enriched in saturated phospholipids and cholesterol (Lingwood and Simons, 2010; Sezgin et al., 2017). These microdomains, termed as lipid rafts, exist as distinct liquid-ordered phases that float freely as stable entities in the liquid-disordered matrix of the plasma membrane. Lipid rafts can lead to a heterogeneous distribution of proteins in the membranes by recruiting them to variable extents (Figure 1C) (Li L. et al., 2020a; Li et al., 2021c). The surface area and length of protein transmembrane domains as well as protein palmitoylation are the major factors determining the affinity of membrane proteins for lipid rafts (Lorent et al., 2017). It is generally believed that lipid rafts, serving as signaling platforms, can facilitate protein-protein interactions on the same membrane by virtue of spatial proximity of participating components. For intercellular receptor-ligand binding, *in situ* experimental studies have reported that lipid rafts help the binding of TCR to pMHC anchored to antigen-presenting cell membranes (Anderson and Roche, 2015). Disrupting the rafts in T cell membrane *via* cholesterol depletion with methyl-beta-cyclodextrin (MβCD) directly reduces the binding affinity *K*_a_, but increases the off-rate *k*_off_ of TCR-pMHC interaction (Huang et al., 2010). To further uncover the mechanism underlying the effect of lipid rafts on the intercellular receptor-ligand binding kinetics, these microdomains are incorporated into the coarse-grained MC model described above (Li et al., 2021b; Li et al., 2021c). The lipid rafts are modeled as dynamic patches experiencing the contact energy with their nearest neighbors. Similarly, the spatial distribution of lipid rafts is also described by the composition field. The association of receptor and ligand with lipid rafts is taken into account by introducing the coupling energy, i.e., the raft affinity to adhesion proteins. Simulation results from this multicomponent membrane system with biologically relevant parameters consistently show that the preferential partitioning of membrane-anchored receptor and ligand proteins in the lipid rafts significantly increases the binding affinity of those proteins, depending strongly on the properties of lipid rafts such as area fraction, size and the affinity of rafts to the proteins (Li et al., 2017b; Li et al., 2017c; Li et al., 2018a; Li et al., 2021d). This enhancement is traced back to the entropy gain of the membranes resulting from raft-induced protein aggregation. Contrary to the case of homogeneous membranes where the binding of the anchored receptor and ligand molecules is weakened by the shape fluctuations of the membranes, the membrane roughness actually functions as a positive regulator for the binding in collaboration with lipid rafts. The bending rigidity contrast between the lipid rafts and liquid-disordered domains further helps the aggregation of proteins and therefore facilitates the binding (Li et al., 2017b; Li et al., 2021b). These studies suggest that cells might regulate the binding kinetics of membrane-anchored receptors and ligands by modulating raft characteristics under physiological conditions.

### Length Difference of Proteins

Length difference of proteins imposes a steric barrier for the bond formation and affects protein distribution, thus affecting the intercellular receptor-ligand binding kinetics. There are a variety of membrane-anchored receptor and ligand molecules with different lengths in adhesion zone (Figure 1D). For example, important receptor-ligand complexes in the T cell adhesion to antigen-presenting cells include the TCR-pMHC complex with a length of about 15 nm, the CD2-CD48 complex with approximately the same length as TCR-pMHC complex, and the LFA1-ICAM1 complex with a length of about 40 nm (Li et al., 2021a). The difference in lengths between the shorter and longer receptor-ligand complexes forms a steric barrier for the formation of the two types of protein complexes, thus affecting their binding kinetics. Milstein et al. utilized the planar bilayer system to examine the effect of difference in complex lengths by increasing the size of ligand CD48, and found that nanoscale increase in the length of CD2-CD48 complexes increases the average intermembrane spacing and decreases the adhesion strength of the receptor-ligand interaction (Milstein et al., 2008). Meanwhile, the difference in lengths between the shorter and longer receptor-ligand complexes can lead to a membrane-mediated repulsion between them because the lipid membranes have to be bent to compensate for the length mismatch, which costs elastic energy (Li et al., 2021a). Milstein et al. observed that both CD2-CD48 variant complexes with elongated ligand segregate from the CD2-CD48 wildtype complexes for specific protein densities (Milstein et al., 2008). Experimental studies of a T cell adhering to supported bilayer with pMHC and ICAM1 have also showed that intercellular protein complexes with different lengths segregate and form characteristic special patterns with a central domain of TCR-pMHC complexes surrounded by a ring-shaped domain of LFA1-ICAM1 complexes for a range of protein densities and affinities (Grakoui et al., 1999; Hammer et al., 2019). Of note, several other mechanisms based on the active transport by actin cytoskeleton, signaling, pre-clustering of TCRs have also been proposed for the formation of the bull’s-eye pattern during T-cell adhesion (Dustin and Cooper, 2000; Choudhuri and Dustin, 2010; Lillemeier et al., 2010; Hammer et al., 2019; Li et al., 2021a). These mechanisms certainly do not need to be mutually exclusive, but instead work together to contribute to the pattern formation. MC simulations and statistical-mechanical calculations for two types of anchored ligands binding to different cell receptors showed that coexistence of domains enriched in the shorter and longer receptor-ligand complexes requires equal effective binding strengths (Rozycki et al., 2010). This length difference-induced protein aggregation will locally affect the binding kinetics of each type of receptor and ligand (e.g., increase the on-rate constant) due to cooperative binding discussed above.

In addition to specific binders, the cells are also covered with anchored polymers or glycoprotein (Figure 1D). These repulsive repellers protruding from both membranes form a protective barrier, the glycocalyx, and can impose an additional steric barrier for the formation of receptor-ligand complexes with a length shorter than that of repellers. Interestingly, the composition and expression level of glycocalyx change markedly with cell fate transitions and cell type. Mulivor and Lipowsky (2002) experimentally observed that removal of the glycocalyx with heparinase increases leukocyte-endothelial cell adhesion, leading to the conclusion that the glycocalyx presents a physical barrier to adhesion and that the shedding of glycocalyx during natural activation of endothelial cells may be an essential part of the inflammatory response. Lorz et al. (2007) analyzed the adhesion of giant vesicles decorated with sialyl-Lewis^X^ ligands and lipopolymers to E-selectin-functionalized substrate by means of reflection interference contrast microscopy and found that the lipopolymers decrease the affinity of receptor-ligand binding. Paszek et al. (2014) found that the overall rate of integrin bond formation reduces in the presence of the glycocalyx. Recent simulation and theoretical studies investigate the binding kinetics of a few and a large number of bonds in the presence of the glycocalyx, representing the cases of initial and mature stages of cell adhesion, respectively (Xu et al., 2016). It is found that the glycocalyx affects the binding kinetics differently for the two cases in the force loading case. More specifically, increasing thickness and stiffness of the glycocalyx decreases the binding affinity for a few bonds, but has negligible effect on the affinity for a large number of bonds. Meanwhile, the thicker glycocalyx is shown to facilitate the clustering of receptors, consistent with the experimental results by Paszek et al. (2014), showing the cancer glycocalyx enhances integrin clustering into focal adhesions and promotes cell growth and survival. These results suggest that the glycocalyx are attractive targets for therapeutic interventions that aim at mediating receptor-ligand interaction.

Studies on the effects of protein-membrane interaction on the receptor-ligand binding kinetics have provided routes and strategies for novel therapies. Extensive studies have revealed that the lipid rafts are involved in a variety of diseases, such as cancer, viral infection, neurodegenerative diseases (e.g., Alzheimer, Parkinson and Prion diseases), immunological diseases (e.g., systemic lupus erythematosus) (Simons and Ehehalt, 2002; Vona et al., 2021). In view of the critical role of lipid raft in cell adhesion and migration by regulating intercellular receptor-ligand binding, therapeutic strategies targeting lipid raft by modulating cholesterol have opened exciting new avenues for cancer prevention and treatment (Vona et al., 2021). Lipid rafts also contribute to the binding and entry of different viruses to host cells, including human immunodeficiency virus (HIV) and coronaviruses. Take the syndrome coronavirus-2 (SARS-CoV-2) for example, it reveals that lipid rafts provide a functional platform able to concentrate angiotensin-converting enzyme-2 (ACE-2), the main receptor for SARS-CoV-2, on the host cell membrane, which facilitates the interaction of ACE-2 with the spike protein on viral envelope (Sorice et al., 2021). Lipid raft disruption by drugs (e.g., stains and cyclodextrins) can lead to reduced SARS-CoV-2 infectivity. The effect of lipid rafts targeting drugs on the infectious process of coronavirus introduces a new potential task in the pharmacological approach against coronavirus that currently ravages the world. Glycocalyx has also been an attractive target for therapeutic interventions due to its implication in the platelet and leucocyte adhesion, inflammatory processes by affecting intercellular receptor-ligand binding. Therapeutic strategies designed to restore the glycocalyx have led to promising results both in the treatment of chronic vascular disease and in an acute critical care setting (Becker et al., 2010). In addition, editing the cancer cell glycocalyx with an antibody-enzyme conjugate to intervene the intercellular receptor-ligand binding between natural killer cell and cancer cell is shown to enhance tumor cell susceptibility to antibody-dependent cell-mediated cytotoxicity (ADCC), thus providing a promising approach to cancer immune therapy (Xiao et al., 2016). Together, an in-depth study regarding the effect of protein-membrane interaction on the receptor-ligand binding kinetics will further provide potential therapeutic strategies and targets for disease prevention and treatment.

## Biomechanical forces

**FIGURE 2:**
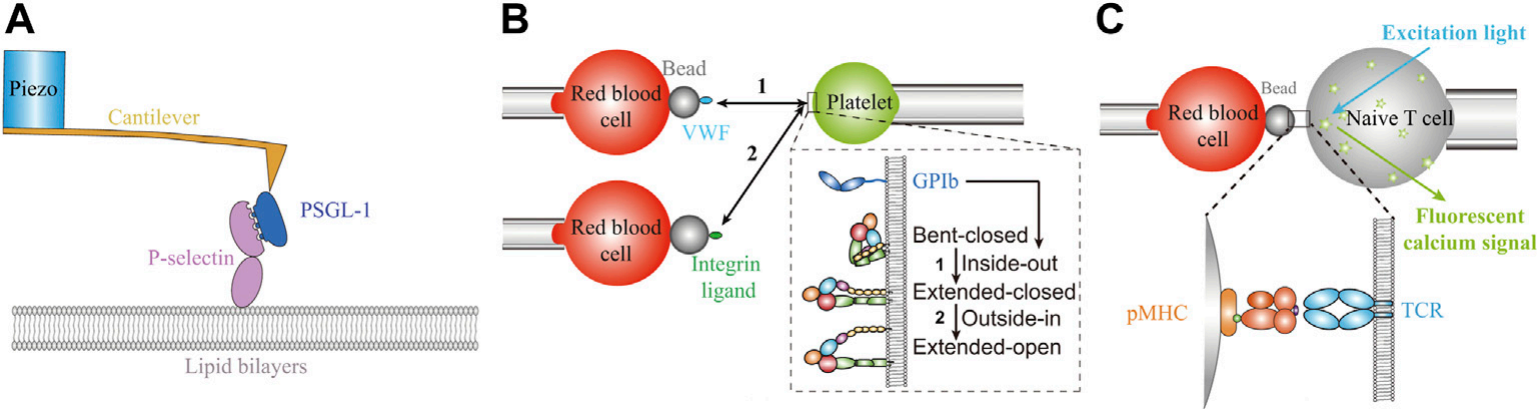
Biomechanical regulations of membrane receptors’ functions revealed by SMFS techniques. **(A)** AFM dissecting biomechanical regulations on the interaction of P-selectin and PSGL-1 [Adapted from Ref. (An et al., 2017)]. **(B)** Dual BFP system revealing biomechanical-chemical coupling signal transduction circuits on platelet. **(C)** Fluorescent imaging integrated BFP system digitalizing the triggering threshold of TCR [Adapted from Ref. (An et al., 2017)].

Benefitting from the development of biomechanical techniques, researches on mechanobiology have leaped ahead in the past decades (Su and Ju, 2018; Zhu et al., 2019a; Wang et al., 2022). Biomechanical tools, such as traction force microscopy, micropillar array and DNA force probe, have definitely confirmed the existence of biomechanical forces actively exerted by single cells to their binding partners through receptor-ligand interactions (Wang and Ha, 2013; Bashour et al., 2014; Liu et al., 2016; Colin-York et al., 2019; Ma et al., 2019). Further, single-molecule force spectroscopy (SMFS) techniques, mainly including atomic force microscopy (AFM), optical tweezers (OT), magnetic tweezers (MT) and biomembrane force probe (BFP), resolve biomechanical regulatory mechanisms of intercellular receptor-ligand binding kinetics in single-molecule level (Neuman and Nagy, 2008; Brenner et al., 2011; Liu et al., 2015). These novel techniques have revealed crucial biomechanical regulatory effects on intercellular receptor-ligand binding kinetics, which is an unachievable task for ensemble protein based biochemical methods, such as SPR (Liu et al., 2015; Zhu et al., 2019a). Force-dependent binding kinetics were firstly proposed by Bell in 1978, demonstrating that mechanical force exponentially accelerates molecular bond dissociation (slip bond) (Bell, 1978). Along with the development of SMFS techniques, a series of catch bonds, whose dissociation rates are conversely slowed down by mechanical forces, are found to play essential roles in cellular activities, such as trafficking, adhesion and antigen recognition (Marshall et al., 2003; Chen et al., 2010; Wu et al., 2019). For example, catch bond is directly observed by AFM measurement of P-selectin/P-selectin glycoprotein ligand-1 (PSGL-1) interaction, correlating with leukocyte adhesion to vascular surfaces under dynamic shear stress applied by blood flow (Figure 2A) (Marshall et al., 2003). The average lifetime (1/*k*_off_) ranking of different TCR-pMHCs bonds at zero force is completely reversed by 10 pN force due to catch bond behavior of agonist pMHCs, and the lifetime ranking under 10 pN force perfectly matches peptide potency, suggesting a crucial role of biomechanical force during TCR-based antigen recognition process (Liu et al., 2014). In addition, “ideal bonds”, whose dissociation rates are insensitive to the sustaining forces, are also found in cadherin adhesion (Rakshit et al., 2012). Noting that these crucial regulatory mechanisms are undetectable by conventional biochemical methods, which measure receptor-ligand binding kinetics in force-free manners (Liu et al., 2014; Wu et al., 2019).

Moreover, in contrast to purified protein based biochemical methods, live cell based SMFS techniques detect membrane receptor-ligand binding kinetics in a more physiological-relevant cellular microenvironment and provide opportunities for dissecting the biomechanical-chemical coupling signal transduction circuits (An and Chen, 2021). As a representative example, integrins can adopt three kinds of conformational states: bent-closed, extended-closed and extended-open conformations, which are strongly associated with their ligand binding strength (Chen et al., 2010; Chen et al., 2019). The equilibrium of the three states can be altered by biomechanical forces induced by ligand binding (outside-in), as well as chemical inside-out signaling events (Figure 2B) (Chen et al., 2010; Chen et al., 2012; Springer and Dustin, 2012; Li J. et al., 2017a; Chen et al., 2019). The interaction between VWF and GPIb under biomechanical tension induces platelet integrin conformational shifts from bent-closed (low-affinity) state to extended-closed (intermediate-affinity) state, as an inside-out signaling pathway for integrin activation (Figure 2B) (Ju et al., 2016; Chen et al., 2019). Further mechanical affinity maturation of the intermediate integrins demands outside-in signaling, with ligand binding under biomechanical tension as a requirement (Figure 2B) (Chen et al., 2019). Based on this biomechanical signal transduction circuit, integrin functions as a mechanosensor to mediate platelet adhesion and aggregation processes (Chen et al., 2019). In this regard, live cell based SMFS techniques outperform conventional biochemical methods in revealing the mechano-chemistry of membrane receptor-ligand binding and dissecting their functional mechanisms (Chen et al., 2010; Chen et al., 2012; Chen et al., 2019).

The incorporation of fluorescent spectroscopy into SMFS techniques further allows correlating intercellular receptor-ligand binding kinetics with intracellular signaling cascades, thereby resolving the transmembrane signaling transduction mechanisms of membrane receptors (Kim et al., 2009; He et al., 2012; Liu et al., 2014; Hu and Butte, 2016; Ju et al., 2016; Feng et al., 2017; Brazin et al., 2018). For example, fluorescent imaging integrated BFP successfully quantified the relationship between force-regulated bond lifetimes of intercellular receptor-ligand interactions and intracellular C_a_^2+^ signaling, revealing the triggering mechanisms of crucial membrane receptors on T cells and platelets (Liu et al., 2014; Ju et al., 2016). For TCR-pMHC interactions, catch bonds potentiate their bond lifetimes and reach maximum at ∼10 pN, where C_a_^2+^ responses are also strongest (Liu et al., 2014). Detailed analyses of the series binding-dissociation dynamics with the concurrent fluorescent C_a_^2+^ signals suggest that T cells exhibit C_a_^2+^ signals only when accumulated bond lifetime exceeds 10 s during the first 60 s, digitalizing the threshold for TCR triggering (Figure 2C) (Liu et al., 2014). Similarly on platelet, GPIb-VWF interactions under stretching forces induce cooperative unfolding processes of two separate domains in GPIb, determining the intensity and type of C_a_^2+^ signals in platelets and transducing extracellular biomechanical stimuli into intracellular biochemical signaling cascades (Ju et al., 2016). More comprehensive biomechanical regulations on membrane receptor-ligand binding kinetics have been summarized in published review articles (Liu et al., 2015; Zhu et al., 2019a; Zhu et al., 2019b; An and Chen, 2021).

In most of the aforementioned single-molecule researches, receptor-ligand bond lifetimes are collected under constant forces (known as force-clamp assay) to reveal biomechanical regulatory mechanisms of intercellular receptor-ligand binding kinetics. However, biomechanical forces sustained by intercellular receptor-ligand bonds are dynamic *in situ*, rather than constant. Experimentally, cytoskeletal forces transducing to and exerting on intercellular receptor-ligand bonds are revealed to be dynamic by traction force microscopy (Colin-York et al., 2019). Moreover, a “motor-clutch” model has been proposed to theoretically characterize the dynamic traction forces induced by myosin movements (Chan and Odde, 2008). The cyclic traction forces sustained by the membrane receptor-ligand molecular bonds depend on substrate stiffness, where softer substrates give rise to larger traction forces (Chan and Odde, 2008; Elosegui-Artola et al., 2016). It has been found that the dynamic force waveforms with different force application histories experienced by the receptor-ligand bonds would affect their dissociation rates and determine membrane receptors’ functions (Kong et al., 2013; Zhu et al., 2019b). For example, cyclic forces applied to integrin-ligand bond result in bond lifetime reinforcement, manifesting a “cyclic mechanical reinforcement” effect (Kong et al., 2013). In this way, the physiologically relevant dynamic forces on intercellular receptor-ligand bonds would potentially enforces more delicate regulations on membrane receptors’ functions. Nevertheless, how to accurately and efficiently investigate the biomechanical force dynamics sustained by *in situ* intercellular receptor-ligand bonds are still problems unresolved.

Complementary to the experiments, all-atom MD simulations have been extensively used to uncover the mechanisms underlying the regulation of biomechanical forces in the receptor-ligand binding kinetics by providing high temporal resolution and atomic details (Hu et al., 2019; Wu et al., 2019; Fan et al., 2022). In contrast to the coarse-grained MD model, all-atom MD simulation method models the native structure of a protein at atomic detail, and all-atom force fields are used for every type of atoms in the receptor-ligand binding system, including hydrogen. Atomic trajectories are then calculated by solving Newton’s Laws of motion. Compared to the coarse-grained simulation method, the computational expense of explicitly modeling every atom limits the atomistic MD simulation to a timescale up to tens of nanoseconds. Fan et al. performed atomistic MD simulation to study the force-strengthened binding affinity and bond lifetimes of NKG2D and MICA. They found that additional hydrogen bond forms at the NKG2D-MICA binding interface in response to the mechanical force. The force-induced ligand conformational changes impede MICA dissociation under force, thus illuminating the molecular basis for this force-strengthened NKG2D-MICA binding (Fan et al., 2022). Similarly, the force-induce formation of additional hydrogen bonds also occurs at the TCR and pMHC binding interface, which contribute to TCR-pMHC catch bonds and T cell activation (Wu et al., 2019). This force-induced conformational changes in pMHCs help to explain why the T cell-based immunotherapies do not work for some cancer patients (Wu et al., 2019). These atomistic molecular dynamics studies definitely provide insights into the detailed molecular mechanisms of receptor-ligand binding, potentially aiding the design of pharmaceuticals.

Resolving the biomechanical regulatory mechanisms of intercellular receptor-ligand binding kinetics would further contribute to biomedical applications. Immunotherapies, such as monoclonal antibodies, bi-/tri-specific antibodies, CAR-T cells, and TCR-T cells, have efficiently revolutionized cancer treatment (Melero et al., 2007; Rapoport et al., 2015; Garber, 2018; Brinkmann and Kontermann, 2021; Seung et al., 2022). The binding affinity of these reagents versus their respective targets is one of the most instructive parameters in their screening and optimization processes (Labrijn et al., 2019; Staflin et al., 2020; Choe et al., 2021). Although current methodologies, such as SPR, yeast display etc., have efficiently filtered low-affinity candidates, the effects of physiological-relevant biomechanical forces on the expected interactions have been neglected (Malmqvist, 1993; Hoogenboom, 2005; Yang et al., 2016; An et al., 2020; Li R. et al., 2020b). Immune checkpoint blockade monoclonal antibodies are assumed to block inhibitory immune receptors in a soluble (force-free) state. However, *in vivo* imaging assay suggests that myeloid cells capture programmed cell death protein-1 (PD-1) antibody through Fcγ receptor-Fc interactions soon after injection, linking PD-1 expressing T cells to another cell and thereby sustaining biomechanical forces similar to membrane-anchored receptor-ligand interactions (Arlauckas et al., 2017). Thus, the blocking effects of the monoclonal antibodies would rely on not only their force-free affinity but the off-rate under biomechanical forces (An et al., 2020). In this regard, BFP-based force-dependent *k*_off_ measurements of three approved PD-1 antibodies versus PD-1 have been found to outperforms SPR measurements in correlating with their clinical responses (An et al., 2020). The scenarios are similar in bi-/tri-specific antibodies, CAR-T cells, and TCR-T cells, where the expected effective molecular bonds also sustain biomechanical forces. As aforementioned, the average TCR-pMHC bond lifetime ranking can even be reversed by biomechanical forces (Liu et al., 2014). Therefore, taking into account the regulatory effects of biomechanical forces is promising in further optimizing the screening process and improving clinical responses of the immunotherapeutic candidates. Moreover, pathological stiffness alterations have been reported in many diseases, not restricted to cancer (Wuerfel et al., 2010; Tian et al., 2015; Liu et al., 2017). The stiffness alterations affect the biomechanical forces exerting on membrane receptor-ligand bonds (Chan and Odde, 2008; Lei et al., 2021). The stiffness of cancer cells can even affect the response of immunotherapies (Lei et al., 2021; Tello-Lafoz et al., 2021; Tello-Lafoz et al., 2022). Therefore, investigating the biomechanical regulations on intercellular receptor-ligand binding kinetics in depth would provide new strategies for biomedical applications in the near future.

## Bioelectric Microenvironment

**FIGURE 3:**
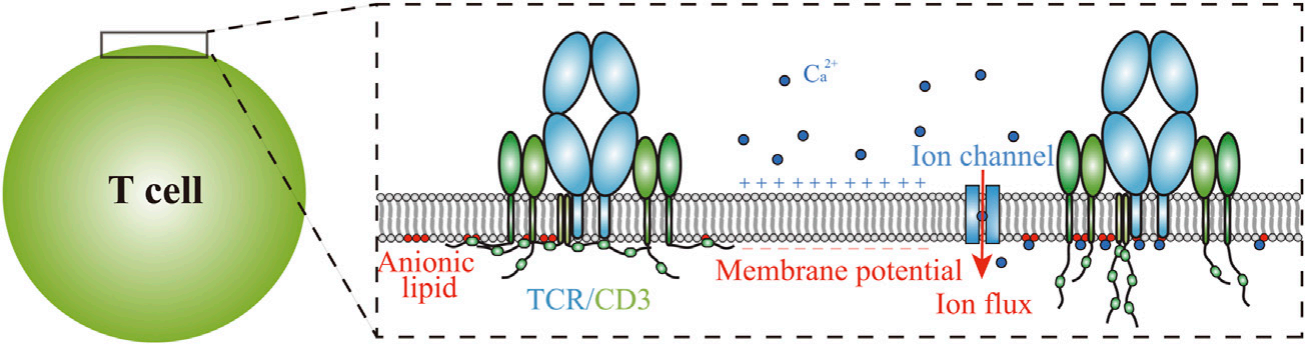
Bioelectric cues surrounding TCRs on T cell membranes. The bioelectric cues that potentially affect TCR functions include membrane potential, ion flux and anionic lipids in the inner cell membranes. Ca^2+^ flux induced by TCR triggering impedes the electrostatic interaction between anionic lipids and the basic rich regions in CD3 tails.

Bioelectric cues surrounding membrane receptors, mainly including membrane potential, charged lipid components, ion flux, etc. (Figure 3), are also crucial biophysical regulators for cells throughout cell lifespan, e.g., modulating key cellular activities including proliferation, differentiation and morphological alterations (Yang and Brackenbury, 2013; Chang and Minc, 2014; Zhou et al., 2015; Boedtkjer and Pedersen, 2020). Especially for neurons, their neuronal activities including neuronal synapse formation, plasticity, maturation, elimination and neuronal excitability are all controlled by dynamic action potential and spontaneous neurotransmitter release (Chubykin et al., 2007; Flavell and Greenberg, 2008; Catterall, 2010; Epsztein et al., 2011; Kwon et al., 2012; Lee et al., 2012; Peixoto et al., 2012; Bian et al., 2015; Sudhof, 2018). The investigations on molecular mechanisms underlying these bioelectrical regulations are mostly confined to ion channels and intracellular signaling cascades (Flavell and Greenberg, 2008; Catterall, 2010; Zhou et al., 2015). Among these studies, the structural and functional mechanisms of voltage-gated ion channels have been thoroughly resolved, where the transmembrane helices in the ion channels perform allosteric alterations in response to membrane potential changes, known as ‘helix-sliding’ (Catterall, 2010). However, whether and how non-ion-channel membrane receptors, as the sensors of cells to collect outside stimuli by interacting with ligands anchored in apposing membrane, response to bioelectric alterations and then adjust their binding kinetics with ligands remain largely unknown.

There are indications that membrane potential directly regulates receptor-ligand binding kinetics. Take for example the G-protein coupled receptors (GPCRs), which have been widely investigated due to their prevalent expression patterns and paramount biomedical significance (Hopkins and Groom, 2002; Mahaut-Smith et al., 2008; Vickery et al., 2016). Similar to aforementioned voltage-gated ion channels, a number of GPCRs have been shown to be membrane potential-sensitive, and the binding kinetics of soluble ligand to GPCRs are demonstrated to be membrane potential-dependent (Ben-Chaim et al., 2006; Mahaut-Smith et al., 2008). All-atom MD studies revealed that membrane potential changes induce conformational alterations inside the transmembrane domains of GPCRs, which constructs the ligand binding site and the intracellular effector binding site, accordingly altering their ligand binding affinity, as well as the signaling cascades (Ben-Chaim et al., 2006; Rinne et al., 2015; Vickery et al., 2016). In addition, the effects of membrane potential on GPCRs binding are GPCR-specific and ligand-dependent, indicating delicate modulations of GPCRs to cellular activities (Ben-Chaim et al., 2006; Navarro-Polanco et al., 2011; Rinne et al., 2015). More importantly, the membrane potential-induced conformational change that underlies receptor binding ability would potentially be utilized as a general principle to regulate the intercellular binding kinetics of adhesion GPCRs to ligands anchored in apposing membrane (Vizurraga et al., 2020).

It should be noted that bioelectric cues surrounding membrane receptors are actually interconnected. One of the representative examples is that membrane potential modulates the distribution of anionic lipids, such as phosphatidylserine (PS) and phosphatidylinositol 4,5-biphosphate (PIP2) (Zhou et al., 2015). These anionic lipids not only affect the localization of cytosolic proteins, such as K-Ras and synaptotagmin-1, but potentially lead to the aggregation of membrane receptors with positively charged regions in their cytoplasmic domains (Shi et al., 2013; Park et al., 2015; Zhou et al., 2015). These distributional alterations can affect intercellular receptor-ligand binding affinity through cooperative binding and accordingly modulate receptor triggering (van der Merwe and Dushek, 2011; Hu et al., 2013). Besides, the electrostatic interactions between anionic lipids and positively charged cytoplasmic tails of membrane receptors are further modulated by ion fluxes (Shi et al., 2013). For example, local C_a_^2+^ concentration enhancement induced by TCR triggering shields the anionic lipids and releases CD3 tails from membrane to facilitate its tyrosine phosphorylation (Figure 3) (Shi et al., 2013). Although these conformational changes occur mainly in the intracellular domain of membrane receptors, the possibility that the intercellular receptor-ligand binding kinetics undergoes allosteric modulations cannot be excluded (Hong et al., 2018). The reasonable scenario is that membrane receptors orchestrate different bioelectric cues to finely tune their ligand binding kinetics and thereby modulate downstream signaling cascades transduced across cell membranes, needs to be further verified.

Although studies regarding bioelectric regulations on intercellular receptor-ligand binding kinetics are still limited due to the lacking of efficient tools, the aforementioned researches provide promising pathways to achieve a significant breakthrough in biomedical applications. For GPCR-targeting medicines, valuable information on voltage-induced conformational changes in GPCRs can be exploited to study novel therapeutic pathways and contributes to biomedical treatments, such as cardiovascular drug development (Navarro-Polanco et al., 2011; Vickery et al., 2016). In view of the aforementioned bioelectric modulation that positively charged CD3 tails are shielded by anionic lipids in the inner cell membranes of T cells in resting states, new strategies for potentiating CAR-T cell persistence have been proposed, where the basic rich sequence of CD3 tail is incorporated into CAR-T design (Shi et al., 2013; Wu et al., 2020). Research on the effect of bioelectric microenvironment on the intercellular receptor-ligand binding is just unfolding. Further resolving the bioelectric regulatory mechanisms of intercellular receptor-ligand binding kinetics would undoubtedly inspire new strategies for biomedical applications.

## Conclusion and Perspective

Cells communicate with their immediate neighbors by intercellular interactions of membrane-anchored receptors and ligands to govern numerous biological processes, such as signal transduction, tissue formation, immune responses, as well as cancer invasion and metastasis (Krobath et al., 2009; Briquez et al., 2020; Cho et al., 2021; Li et al., 2021d; Li Y. et al., 2021e). The two-dimensional receptor-ligand interactions have attracted extensive attention in the past decades, due to their great potential to stimulate new strategies in drug design and improve disease prevention and treatment. The key parameters quantifying the intercellular receptor-ligand interactions are their binding kinetics. In sharp contrast to the binding in solution, the intercellular binding kinetics appear to depend strongly on the cellular microenvironment, requiring more in-depth investigation to elucidate the regulatory mechanisms. This review summarizes the advances regarding the regulatory effects on the intercellular receptor-ligand binding kinetics mainly from three aspects: 1) protein-membrane interaction, 2) biomechanical force, and 3) bioelectric microenvironment. We introduce modeling methods and experiment technologies developed for dealing with issues at different scales and provide insights into the underlying mechanisms. Meanwhile, we outline future directions to advance the fields of intercellular receptor-ligand binding kinetics and drug discovery. For example, the dynamic nature of biomechanical forces sustained by the intercellular receptor-ligand bonds under physiological conditions needs to be accurately quantified, and how the dynamic forces affect the intercellular binding kinetics also needs to be further illuminated. In addition, the role of bioelectric microenvironment in intercellular interactions has become a pressing issue to be solved. These prospective studies would contribute to identifying potential new strategies for drug development and disease therapy.

In fact, these regulatory factors for the intercellular receptor-ligand binding kinetics, which are investigated separately in general, are not mutually exclusive but instead are closely interrelated. For example, mechanical tension within the axons contributes to clustering of neurotransmitter vesicles at presynaptic terminals, which is implicated in neurotransmission efficiency and electrical activity at the synapse (Siechen et al., 2009). In addition, the bioelectric microenvironment surrounding membranes can change their mechanical property (e.g., bending rigidity) (Faizi et al., 2019), which in turn affects the thermal shape fluctuations of flexible membranes, thus leading to the alternation of intermembrane local separation and dynamic force sustained by the intercellular receptor-ligand bonds. To obtain a comprehensive understanding of intercellular receptor-ligand binding kinetics under physiological conditions for the discovery of more effective drugs, further studies on coupling effect of regulatory factors on the intercellular binding kinetics based on more complicated multiparameter systems are needed. Coupled with innovations in technology, the results of future studies will keep contributing to the rational design of clinically effective drug and promoting the transition from a promising field of intercellular receptor-ligand binding kinetics to medical application.
